# Supplementary material for: Deterministic positioning and alignment of a single-molecule exciton in plasmonic nanodimer for strong coupling
Source: Nat Commun. 2024 May 16;15:4103. doi: 10.1038/s41467-024-46831-6 (PMC11099047; doi:10.1038/s41467-024-46831-6)
Supplement: Supplementary file 1 — Supplementary Information [file 41467_2024_46831_MOESM1_ESM.pdf]

Supplementary Information for

Deterministic positioning and alignment of a single-molecule exciton in plasmonic nanodimer for strong coupling

Renming Liu[1,2,#,*], Ming Geng[1,#], Jindong Ai[1,#], Xinyi Fan[1], Zhixiang Liu[1], Yu-Wei Lu[3], Yanmin Kuang[1], Jing-Feng Liu[4*], Lijun Guo[1*], and Lin Wu[5,6*]

[1]School of Physics and Electronics, International Joint Research Laboratory of New Energy Materials and Devices of Henan Province, Henan University, Kaifeng 475004, China

[2]Institute of Quantum Materials and Physics, Henan Academy of Sciences, Zhengzhou 450046, China

[3]Quantum Science Center of Guangdong–Hong Kong–Macao Greater Bay Area (Guangdong), Shenzhen 518045, China

[4]College of Electronic Engineering, South China Agricultural University, Guangzhou 510642, China

[5]Department of Science, Mathematics and Technology, Singapore University of Technology and Design, 8 Somapah Road, Singapore 487372, Republic of Singapore.

[6]Institute of High Performance Computing, Agency for Science, Technology, and Research (A*STAR), 1 Fusionopolis Way, No. 16-16 Connexis, Singapore 138632, Republic of Singapore.

[#]These authors contributed equally: Renming Liu, Ming Geng, and Jindong Ai.

[*]liurm@henu.edu.cn; liujingfeng@scau.edu.cn; juneguo@henu.edu.cn; lin_wu@sutd.edu.sg

Contents

Supplementary Note 1: Evidences for the formation of CB[7]@single MB emitters

Supplementary Note 2: Numerical simulations

Supplementary Note 3: Calculations of mode volume (V_m), Purcell factor (F_p), and coupling constant (g_0)

Supplementary Note 4: Quantum mechanical model for describing plasmon-emitter strong coupling

Supplementary Note 5: Photobleaching of the strongly coupled AuND/CB[7]@single MB systems

Supplementary Note 6: Polarization dependence of plexcitonic properties on strongly coupled AuNDs

Supplementary Note 7. Materials and Methods

Supplementary Figures 1-21

Supplementary Tables 1 and 2

Supplementary References 1-25

Supplementary Note 1. Evidences for the formation of CB[7]@single MB emitters

The encapsulation of a single MB molecule inside the cucurbit[7]uril (CB[7]) can be confirmed by the following pieces of evidence: firstly, the absorption spectroscopy of MB dimers (shown by the small shoulder peak at ~615 nm on the black curve) almost disappears on mixing MB with CB[7] (in a 1:2 molar ratio) (the red curve in Supplementary Fig. 1). Control experiments with the smaller CB[5] molecules (into which MB molecule cannot fit) do not remove this shoulder peak (the dashed red line in Supplementary Fig. 1), ruling out parasitic binding[1]. Secondly, the fluorescence intensity of the MB monomers can be enhanced more than three times when they are embedded in CB[7]s (Supplementary Fig. 2a), arising from the enhancement of the fluorescence quantum yield of these monomers hosted in CB[7] cavities. Thirdly, that the lifetime of the encapsulated MB monomer in CB[7] is significantly prolonged to 2.11 ± 0.02 ns from 0.48 ± 0.01 ns of the bare MB monomer (Supplementary Fig. 2b), agreeing with that observed in the literature[2,3].

Supplementary Note 2: Numerical simulations

Simulation of the electric field (EF) enhancement in the bare AuNDs with different gap distances. The normalized scattering and EF enhancements in the bare AuNDs with different gap distances were calculated using the finite element method (FEM) (Supplementary Fig. 7)). In the calculations, the bare AuNDs were fixed on the surface of the ITO-coated glass substrate, and each AuND was constructed by two Au nanospheres 40 nm in diameter. The gap between the two nanospheres was set at 0.9, 2, 5, and 10 nm, respectively. The ITO-coated glass substrate was simulated by a dielectric layer with a refractive index of 2.0. Considering the environment around the small AuNDs and the surface roughness of the ITO film relative to the small Au nanoparticles, an effective refractive index of 1.26 above the ITO film was utilized in our calculations.

Simulation of scattering spectra for the bare AuNDs with different particle sizes. The scattering spectra of the bare AuNDs with different particle sizes were calculated using the FEM. In the calculations, the bare AuNDs were fixed on the surface of the ITO-coated glass substrate, and each AuND was constructed by two identical Au nanospheres with diameters varying from 30 to 54 nm. The gap distance between the two nanospheres was set at 0.9 nm. The ITO-coated glass substrate was simulated by a dielectric layer with a refractive index of 2.0, and the effective

refractive index of 1.26 above the ITO film was utilized. In addition, taking into account a single CB[7] molecule integrated into the gap of the bare AuND in the experiment, we used a dielectric cylinder with a diameter of 1.5 nm and a length of 0.9 nm (equals the size of the CB[7] molecule) to represent the CB[7] molecule, and the refractive index of this dielectric cylinder was set as 1.45.

Simulations for the absorption cross-section spectra of the coupling components. In the simulations, we used the FEM to calculate the absorption cross-section spectrum of the MB molecule by integrating the total density of power dissipation in the domain of the CB[7]@single MB, i.e., the dielectric cylinder between the two Au nanospheres (the dielectric permittivity of the cylinder was described by the Lorentz model, as we have mentioned in the main text), and then the integral result was normalized to the intensity of the incident field (I_0). Similarly, the absorption cross-section spectrum of the AuND in the hybrid system was calculated by integrating the total density of power dissipation in the domain of the two Au nanospheres (the dielectric permittivity of Au dimer was described by the Brendel-Bormann model). Then, the integral result was also normalized to the intensity of the incident field (I_0).

Supplementary Note 3: Calculations of mode volume (V_m), Purcell factor (F_p), and coupling constant (g_0)

Calculation of V_m and g_0. Since the AuND has a sub-nanometer gap, most of the field $|\mathrm{E}(\mathbf{r},\ \omega)|$ and energy density are confined in the gap between the nanoparticles (Fig. 1e in the main text); the permittivity $\varepsilon(\mathbf{r},\ \omega)$ is still dominated by the refractive index in the gap of the dimer. Therefore, the AuND with a sub-nanometer gap can seem like an optical cavity, and its mode volume V_m can be calculated using[1,4]:

$$V_m(\omega) = \frac{\int \varepsilon(\mathbf{r},\ \omega)\left|\mathrm{E}(\mathbf{r},\ \omega)\right|^2 \mathrm{d}\mathbf{r}}{\max\left[\varepsilon(\mathbf{r},\ \omega)\left|\mathrm{E}(\mathbf{r},\ \omega)\right|^2\right]}\ , \tag{1}$$

via the FEM. To make this equation applicable to plasmons, the term $\varepsilon(\mathbf{r},\ \omega)$ can be modified as[4,5],

$$\varepsilon(\mathbf{r},\ \omega) \rightarrow \mathrm{Re}[\varepsilon(\mathbf{r},\ \omega)]+2\omega\,\mathrm{Im}[\varepsilon(\mathbf{r},\ \omega)]/\beta\ , \tag{2}$$

where $\varepsilon(\mathbf{r}, \omega)$ is the permittivity of the metal at the position of $\mathbf{r}$, β is the plasmonic damping term, and Re[] and Im[] are the real and imaginary parts, respectively.

In V_m calculations, the CB[7] embedded in the gap of the AuND was modeled as a dielectric cylinder (0.75 nm in radius and 0.9 nm in length) with a refractive index of 1.45, and the mesh was set at 0.1 nm in the nanogap. Notice that, due to the significant roughness of the surface of the ITO-coated glass substrate compared to the Au nanoparticle size, an effective refractive index of $n_{eff} = 1.26$ was set above the ITO substrate to ensure the calculated resonant wavelength of the AuNDs agrees with the experimental measurement results under the same structural parameters. Based on the calculated $V_{eff} = V_m \times n_{eff}^2$, the coupling constant, g_0, between the plasmons and a single exciton can be calculated using[5],

$$g_0 = \frac{\hbar\omega_c}{\sqrt{2\hbar\varepsilon_0\omega_d V_{eff}}} \boldsymbol{\mu}_c \cdot \hat{\mathbf{f}}_d(\mathbf{r}_c), \quad (3)$$

where $\hat{\mathbf{f}}_d(\mathbf{r}_c)$ is the normalized EF of the plasmon mode ($\hat{\mathbf{f}}_d(\mathbf{r}) = \mathbf{E}_d(\mathbf{r}) / |\mathbf{E}_d(\mathbf{r})|_{max}$) at the exciton position $\mathbf{r}_c$. In calculating g_0, the transition dipole moment ($\boldsymbol{\mu}_c$) of a single MB-molecule exciton was set at 0.09 e nm, and the $\boldsymbol{\mu}_c$ orientation was set parallel to the plasmonic field $\hat{\mathbf{f}}_d(\mathbf{r}_c)$.

Calculation of V_m using F_p. We have also calculated the V_m of AuND via the following formula[1],

$$F_p = \frac{3}{4\pi^2}\frac{Q}{V_m}\left(\frac{\lambda}{n}\right)^3, \quad (4)$$

F_p is the Purcell factor of a point dipole located in the gap center and oriented along the dimer axis, which can be obtained by[6],

$$F_p(\omega) = \frac{\Gamma}{\Gamma_0} = \frac{\rho_n(\mathbf{r}_c, \omega)}{\rho_n^0(\mathbf{r}_c, \omega)}, \quad (5)$$

where $\rho_n^0(\mathbf{r}_c, \omega)$ is the local density of optical states (LDOS) in free space. It can also be written in terms of the modified spontaneous emission rate Γ of the emitter, divided by its spontaneous emission rate Γ_0 in free space. Our calculations show that the Purcell factor in the gap center is up to F_p ~3.2×10^6 at the resonant wavelength of 659.8 nm (resonant to the exciton transition in

MB molecule, 660 nm) (Supplementary Fig. 1), which can achieve the single-molecule exciton strong coupling in this plasmonic nanocavity[1,6]. Based on this Purcell factor, we can calculate the Q factor, here Q = 12.96 at 659.8 nm, so we can then extract the mode volume at 659.8 nm of V_m ~29 nm^3. It should be noticed that the method of Supplementary Equation (3) is more suitable for calculating the mode volume of a nanocavity excited by the plane wave, as we have utilized in our experiments.

Conversely, the calculated Purcell factor versus wavelength also shows strong dark modes in the higher energies with higher intensities compared to the bright dipolar mode. Unlike that of the single Au (or Ag) nanoparticles[7,8], the energies of the dark plasmonic modes in this AuND are far away from that of the bright dipolar mode, and the transition frequency of the MB molecule (~660 nm), indicating that the interactions between the excitonic transition and the dark plasmonic modes will be in the weak coupling regime. Therefore, the dark plasmonic modes could not result in a pseudo mode hybridizing with the exciton's transition and promoting strong coupling dynamics seen in the spectral Rabi splitting of the bright dipolar mode of an AuND, which is unlike the nanosphere case theoretically demonstrated in ref. 7. It is also an advantage for the AuNDs with sub-nanometer gaps to exploit the strong coupling of single emitters. Despite this, the weak interactions between the dark mode and the emitter may induce a finite frequency shift (Lamb shift) of the emitter transition, which can be reflected by a shift of the splitting valley position in the scattering/absorption spectrum of the AuND-single molecule system[8]. Therefore, one can investigate the Lamb shift effect based on this single-molecule strong coupling system, even though the frequency shift of the emitter in such a sub-nanometer plasmonic gap has a complex mechanism.

Supplementary Note 4: Quantum mechanical model for describing plasmon-emitter strong coupling

This work employs a fully quantum mechanical approach to describe the strong coupling between the plasmon mode and N identical emitters[5]. Specifically, we describe the plasmon mode with bosonic annihilation and creation operators, and the emitter is described as a fermionic system with two possible states (the ground and excited states). The Hamiltonian of this coupling system can be written as[5,9]

$$H = H_0 + H_{int} + H_{decay}\,, \tag{6}$$

where H_0 is the Hamiltonian describing the noninteracting evolution of N emitters and the plasmon mode,

$$H_0 = \varepsilon_{\mathrm{d}} d^+ d + \sum_{i=1}^{N} \varepsilon_{\mathrm{c}i} c_i^+ c_i\,, \tag{7}$$

c_i (c_i^+) is the annihilation (creation) operator for the *i*th emitter with energy of $\varepsilon_{\mathrm{c}i} = \hbar\omega_{\mathrm{c}i}$, and d (d_i^+) is the annihilation (creation) operator of the plasmon mode with energy of $\varepsilon_{\mathrm{c}i} = \hbar\omega_{\mathrm{d}}$. The plasmon-emitter interaction can be modeled by the Hamiltonian:

$$H_{int} = -\sum_{i=1}^{N} g_{\mathrm{dc}i} \left[d^+ c_i + c_i^+ d \right], \tag{8}$$

where $g_{\mathrm{dc}i}$ is the coupling constant between the plasmon mode and the *i*th emitter.

To describe the finite lifetimes of the plasmons and fermions, the Hamiltonian in Supplementary Equation (6) contains the inelastic interactions (H_{decay}) between the coupling subsystems and the environment (a continuum of modes),

$$\begin{aligned} H_{decay} &= \int \mathrm{d}\omega \hbar\omega f_{\mathrm{d}}^+ (\omega)\, f_{\mathrm{d}} (\omega) + \sum_{i=1}^{N} \int \mathrm{d}\omega \hbar\omega f_{\mathrm{c}i}^+ (\omega)\, f_{\mathrm{c}i} (\omega) \\ &- \int \mathrm{d}\omega \left[v_{\mathrm{d}} (\omega)\, f_{\mathrm{d}} (\omega) d^+ + v_{\mathrm{d}}^* (\omega)\, f_{\mathrm{d}}^+ (\omega) d \right] \\ &- \sum_{i=1}^{N} \int \mathrm{d}\omega \left[v_{\mathrm{c}i} (\omega)\, f_{\mathrm{c}i} (\omega) c_i^+ + v_{\mathrm{c}i}^* (\omega)\, f_{\mathrm{c}i}^+ (\omega) c_i \right], \end{aligned} \tag{9}$$

where $f_{\mathrm{c}i}(\omega)$ ($f_{\mathrm{c}i}^+(\omega)$) is the annihilation (creation) operator of the continuum modes that couple to the i th emitter, and the corresponding coupling strength is $v_{\mathrm{c}i}(\omega)$; $f_{\mathrm{d}}(\omega)$ ($f_{\mathrm{d}}^+(\omega)$) is the annihilation (creation) operator of the continuum modes that couple to plasmon mode, the corresponding coupling strength is $v_{\mathrm{d}}(\omega)$.

Assuming that only plasmons coupled efficiently to the external photons, the optical absorption spectrum can be deduced from Zubarev's Green function[10] $\left\langle\left\langle d;d^{+}\right\rangle\right\rangle_{\omega+i0^{+}}$ as,

$$\sigma(\omega)\propto -\mathrm{Im}\left\{\left\langle\left\langle d;d^{+}\right\rangle\right\rangle_{\omega+i0^{+}}\right\} = -\mathrm{Im}\left\{\hbar\omega-\varepsilon_{\mathrm{d}}+\delta\omega_{\mathrm{d}}+i\frac{\Gamma_{\mathrm{d}}}{2}-\sum_{i=1}^{N}\frac{g_i^2\left(1-2n_{\mathrm{c}i}\right)}{\hbar\omega-\varepsilon_{\mathrm{c}i}+\left(1-2n_{\mathrm{c}i}\right)\left(\delta\omega_{\mathrm{c}i}+i\frac{\Gamma_{\mathrm{c}i}}{2}\right)}\right\}^{-1}. \quad (10)$$

If we assume that the identical emitters are indistinguishable from each other, we have for $i=1,2,\ldots N$, $\varepsilon_{\mathrm{c}i}\equiv\varepsilon_{\mathrm{c}},\ g_i\equiv g,\ \Gamma_{\mathrm{c}i}\equiv\Gamma_{\mathrm{c}},\ \delta\omega_{\mathrm{c}i}\equiv\delta\omega_{\mathrm{c}},\ n_{\mathrm{c}i}\equiv n_{\mathrm{c}}$, thus, the extinction spectrum of the strongly coupled system can be written as,

$$\sigma(\omega)\propto -\mathrm{Im}\frac{(\hbar\omega-\hbar\omega_{\mathrm{c}}+i\Gamma_{\mathrm{c}}/2)}{(\hbar\omega-\hbar\omega_{\mathrm{d}}+i\Gamma_{\mathrm{d}}/2)\cdot(\hbar\omega-\hbar\omega_{\mathrm{c}}+i\Gamma_{\mathrm{c}}/2)-Ng_{\mathrm{dc}}^2}, \quad (11)$$

where Γ_{d}, Γ_{c} is the damping linewidth of the plasmon mode and emitter, respectively. In Supplementary Equation (11), we have considered the system to be initially prepared in the ground state $n_c=0$, and the frequency shifts $\delta\omega_{\mathrm{d}}$ and $\delta\omega_{\mathrm{c}}$ have been accounted for by renormalizing the energies of the corresponding states ε_{d} and ε_{c}, respectively.

At resonance $\omega_{\mathrm{d}}=\omega_{\mathrm{c}}$, one can obtain the analytical expressions of the spectral Rabi splitting (SRS, $\hbar\Omega_R$) and its corresponding critical criterion by setting $\mathrm{d}\sigma(\omega)/\mathrm{d}\omega=0$,

$$\hbar\Omega_R=2\sqrt{\sqrt{N}g_{\mathrm{dc}}(1+\Gamma_{\mathrm{c}}/\Gamma_{\mathrm{d}})\cdot(Ng_{\mathrm{dc}}^2+\Gamma_{\mathrm{c}}\Gamma_{\mathrm{d}}/4)^{1/2}-(Ng_{\mathrm{dc}}^2+\Gamma_{\mathrm{c}}\Gamma_{\mathrm{d}}/4)\cdot\Gamma_{\mathrm{c}}/\Gamma_{\mathrm{d}}}\quad \text{if}\quad Ng_{\mathrm{dc}}^2>\frac{\Gamma_{\mathrm{c}}^2}{8(1+\Gamma_{\mathrm{d}}/2\Gamma_{\mathrm{c}})} \quad (12)$$

For single emitter cases, N=1 in the above equations. It should be mentioned that the Supplementary Equations (11) and (12) provide us with an effective and convenient approach to extract the coupling constant based on the experimental measured SRS, Γ_{d} and Γ_{c}; it is not necessary to calculate the mode volume or Purcell factor at first.

Supplementary Note 5: Photobleaching of the strongly coupled AuND/CB[7]@single MB systems

It is well known that when the plasmonic nanoparticle-dye molecule coupling systems experienced laser illumination, photobleaching would occur in the molecules even under a weak laser intensity and a short illumination time[4,5,11]. In our manuscript, we utilized this photobleaching method to destroy the molecule exciton states in the AuND/CB[7]@MB hybrids via the illuminating of a femtosecond laser. It was found that the transparency dips in SRS are remarkably weakened and even completely disappeared under a relatively weak irradiance (10 μW/μm^2 for 40 s) by a 514-nm femtosecond laser (Ti: sapphire laser, Mira 900) (Supplementary Fig. 13). Such an optical bleaching phenomenon in these strong coupling systems indicates that the observed energy splitting in Fig. 2a, b in the main text arises from the MB molecule.

Supplementary Note 6: Polarization dependence of plexcitonic properties on strongly coupled AuNDs

Because the plasmonic field is parallel in AuNDs, it would be more suitable to investigate the polarization-resolved dark field scattering of the strongly coupled AuNDs with emitters. In the following Supplementary Fig. 18, we performed the experiments of polarization-resolved dark field measurements of the strongly coupled AuNDs with MB-molecule excitons. In our measurements, the linear polarizer in the collection path of the microscope was rotated, and a spectrum was collected at each angle from 0° to 90°, corresponding to the longitudinal (horizontal arrow) and transverse (vertical arrow) polarizations (the insets in Supplementary Fig. 18a), respectively. Supplementary Fig. 18 shows two representative cases of the polarization-resolved dark-field scattering for the strongly coupled AuNDs with MB-molecule excitons, in which a progressive emergence of the two plexcitonic states (the UPB and LPB states) as the polarization rotated from transverse (90°) to longitudinal (0°) can be observed. As the polarization angle decreases from 90° to 0°, the coupling between the gap plasmon mode and exciton becomes stronger and stronger. The LPB mode appears gradually on the red side of the exciton transition (660 nm/1.880 eV), which agrees well with the observations reported in the literature[12]. More interestingly, it is also found that the UPB and LPB modes have a different polarization dependence, as shown in Supplementary Fig. 18b, which may effectively manipulate the two hybrid plexcitonic states.

Supplementary Note 7: Materials and Methods

Materials. Gold (III) chloride trihydrate ($HAuCl_4·3H_2O$, > 99%), silver nitrate ($AgNO_3$, 99.8%), sodium borohydride ($NaBH_4$, 99%), L-ascorbic acid ($C_6H_8O_6$, >99%), cetyltrimethylammonium bromide (CTAB, 99%), sodium citrate ($C_6H_5O_7Na_3·2H_2O$, 99%), potassium iodide (KI, 99%), sodium hydrate (NaOH, 97%), Cucurbit[7] urils ($C_{42}H_{42}N_{28}O_{14}$, >99.5%), methylene blue ($C_{16}H_{18}ClN_3S$, 95%) and sodium chloride (NaCl, 99.5%) were purchased from Sigma Aldrich and used as received. In experiments, we used deionized water with a resistivity of 18.25 MΩ·cm, prepared using a Millipore Mili-Q water system.

Synthesis of Au nanoparticles. Highly purified Au nanoparticles were fabricated using our method.[13] Before experiments, all glassware was washed with aquaregia (3:1 ratio by volume of HCl and HNO_3) and rinsed with deionized water several times. Au nanoparticles were grown following a procedure modified from that reported by Millstone et al.[14]. Specifically, Au nanocrystal seeds were prepared by reducing $HAuCl_4$ (0.01 M, 1 mL) with a freshly prepared, ice-cold aqueous $NaBH_4$ (0.1 M, 1 mL) in fresh deionized water (30 mL), followed by rapid a stir mixing for 2 min. The resulting mixture was aged at room temperature (25^{0}C) for 4 hours to allow the hydrolysis of unreacted $NaBH_4$. Finally, the Au nanocrystal seeds were obtained with an average size of ~5 nm (Supplementary Fig. 19) and a plasmon resonance peak at ~518 nm. After the aging period, five growth solutions (A, B, C) were prepared for seed-mediated growth. The first two solutions (A and B) were identical and contained NaOH (0.1 M, 0.05 mL), ascorbic acid (0.1 M, 0.05 mL), KI (0.1 M, 4.5 μL), CTAB (0.05 M, 9.0 mL) and $HAuCl_4$ (10 mM, 0.25 mL). The other solution, C, contained NaOH (0.1 M, 0.25 mL), ascorbic acid (0.1 M, 0.25 mL), KI (0.1 M, 23 μL), CTAB (0.05 M, 45 mL) and $HAuCl_4$ (10 mM, 1.25 mL). Note that all these three solution groups were prepared by adding chemicals in the abovementioned sequence. Au nanoparticle growth was initiated by adding 1 mL of seed solution to solution A, which was gently shaken. After that, 5 mL of growth solution A was quickly added to B, followed by gentle shaking, and 3 mL of solution B was added to the growth solution of C. After the addition, the colors of C changed from clear to deep magenta-purple for 30 minutes for all preparations. Note that this method's original yield of the spherical Au nanoparticles synthesized is ~70%. After growth for 2 hours, NaCl solution (4.0 M, 1.0 mL) was added to the mixture, and the final mixed solution was left undisturbed for 12 h. Then, the supernatant solution was gently transferred to another cleaned beaker by a syringe, and the purity of the spherical Au nanoparticles could be

improved up to ~99%. Note that when we used these purified Au nanoparticles as the seeds to perform the regrowth in the above growth solution, the Au particle size can be effectively enlarged from ~40 to 80 nm. Then, we employed the oxidation etching method of Au^{3+} to smooth the surface of the prepared Au nanoparticles[15], and the smoothed Au nanoparticles with diameters varying from ~30 to 60 nm can be obtained (Supplementary Fig. 4).

The percentage of dimers in the strong coupling. We have measured more than 200 individual AuNDs isolated from the sample mixed with CB[7]@single MB emitters at a concentration of $\bar{n}_{\mathrm{MB}}$ = 0.8 CB[7]@single MB emitters per Au nanoparticle (on average), and it is found that there are about 15% of these measured dimers demonstrate strong coupling. The rest of the measure dimers do not feature energy splitting with and without MB molecules. Such a percentage is understandable and acceptable compared to that (~1%) currently commonly achieved[16]. According to the mole ratio of MB and CB[7] of 1: 2 in the fabrication of CB[7]@single MB emitters, there are at least half of AuNDs (on average) formed via empty CB[7] without MB molecule inside. On the other hand, some dimers (~5%, See Supplementary Fig. 5a-c) formed naturally, and some dimers may have morphological defects that reduce the coupling strength between the gap plasmon mode and exciton. All these factors reduce the percentage of the dimers showing spectral Rabi splitting.

It also should be mentioned that in these strong-coupling cases, the nanodimers featured energy splitting consistent with one MB molecule accounting for about 83.3%, and 13.3% of these strong coupling dimers demonstrate the two-MB strong coupling (Supplementary Fig. 20). A very small part (~3.3%) shows three-MB molecule strong coupling, owing to some cases with non-uniform adsorption of CB[7]@single MB emitters on Au nanoparticles. The quantum steps in Supplementary Fig. 21c demonstrate that most of the individual AuND/CB[7]@single MB hybrids are in the single-exciton strong coupling regime for the sample treated with a low dye concentration ($\bar{n}_{MB}$ = 0.8 CB[7]@single MB emitter per Au nanoparticle).

Supplementary Figures

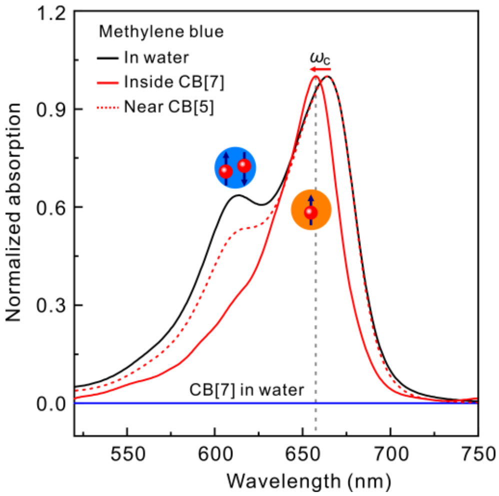

Supplementary Figure 1. Absorption spectra of the methylene-blue (MB) molecules in aqueous solutions with and without CB[n]. Normalized absorption spectra of MB molecules in water (black line), with (solid red) and without (dashed red) encapsulation in cucurbit[n] urils (CB[n]) of different diameters (dashed and solid red lines). The molar ratio of MB and CB[n] is 1:2.

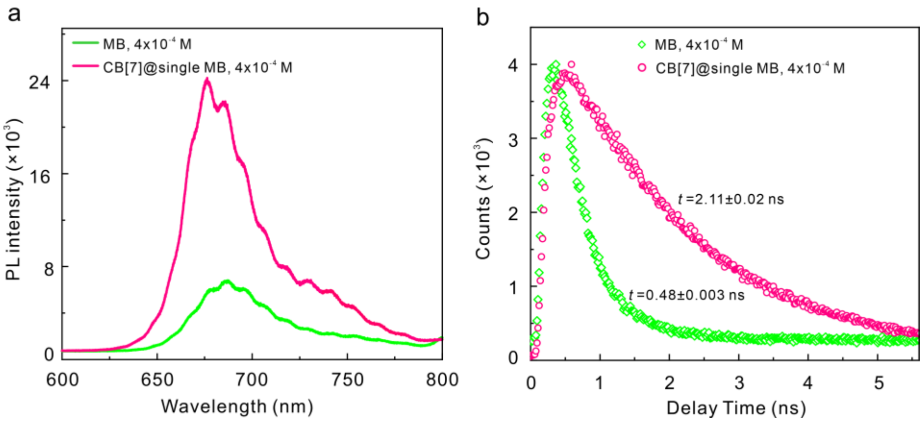

Supplementary Figure 2. Fluorescence properties of the MB molecules in aqueous solutions with and without CB[7]. a PL spectra of MB molecules (4×10^{-4} M) in aqueous solution with (red curve) and without (green curve) CB[7], respectively. **b** Fluorescence decay profiles for MB molecules (4×10^{-4} M) in aqueous solution with (red curve) and without (green curve) CB[7], respectively. MB: methylene-blue.

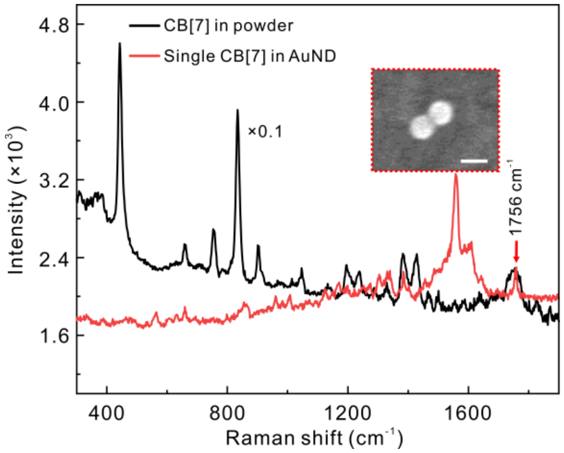

Supplementary Figure 3. Raman and SERS spectra of CB[7] molecules in powder and AuND. Red line: SERS spectrum of a CB[7] molecule embedded in the AuND. Black line: Normal Raman spectrum of the CB[7] molecules in powder. The inset is the SEM image of the measured AuND/single CB[7]. The scale bar is 50 nm. It can be seen that the stretch vibration of the C=O (~1756 cm^{-1}) indeed significantly enhanced compared to other SERS peaks and that in the normal Raman spectrum of the CB[7] molecules in powder, indicating that the binding of CB[7] on gold is through the carbonyl-fringed portals. AuND: Au nanodimer.

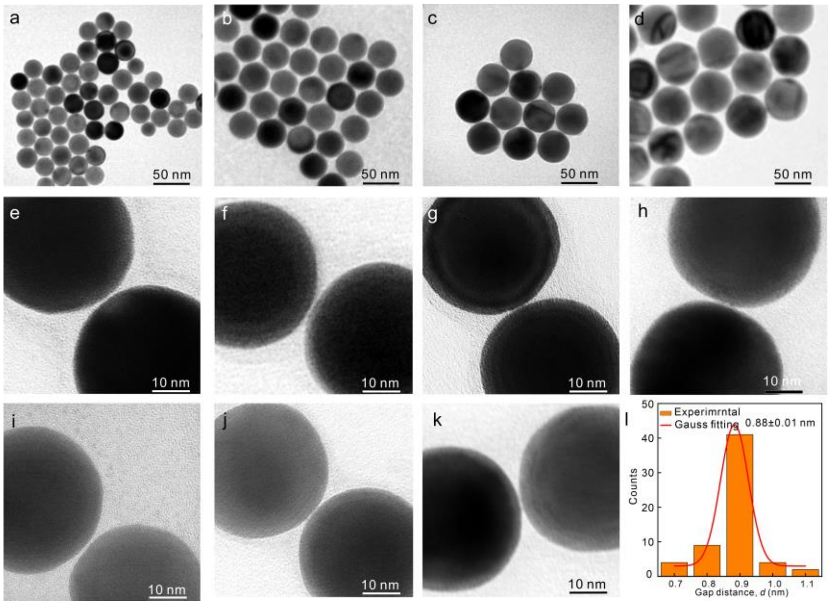

Supplementary Figure 4. Morphology characterization of the Au nanoparticles with different sizes and the AuNDs assembled by CB[7]@single MB emitters. a-d TEM images of the super spherical Au nanoparticles with different diameters of (**a**) 27.0±2.2 nm, (**b**) 38.4±1.8 nm, (**c**) 45.4±0.8 nm and (d) 57.0±2.8 nm, respectively. **e-k** TEM images of the representative

AuNDs with a fixed gap of about 0.9 nm, which are constructed by super spherical Au nanoparticles via single CB[7]@single MB emitters. **l** Statistics of the gap distance (*d*) observed in individual AuNDs hybridized with single CB[7]@single MB emitters, from which one can see that most gaps constructed by CB[7]@single MB emitter are at a fixed value of *d*~0.9 nm, and the mean value of the observed gap distances is *d*~0.88±0.01 nm.

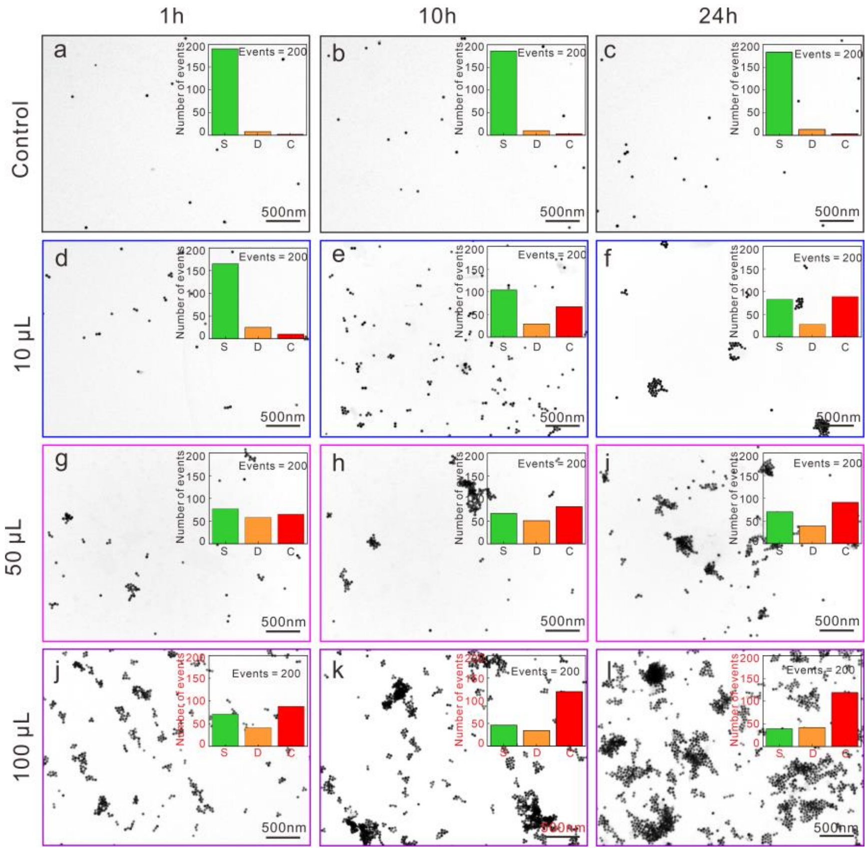

Supplementary Figure 5. Statistics of AuNDs and other aggregations after Au nanoparticles treated by CB[7]@single MB emitters with different concentrations and hybridization times. a-c TEM images of Au nanoparticles isolated from the sample without mixing CB[7]@single MB emitters at different times (after the sample was fabricated 1, 10, and 24 h, respectively). **d-l** TEM images of Au nanoparticles isolated from the samples (2 mL, ~5.0×10^{11}) mixed with CB[7] molecules (6.66×10^{-8} M) with varying volumes of 10, 50, and 100 μL at different hybridization times (1, 10, and 24 h, respectively). The insets are statistical histograms of the single Au nanoparticles, nanodimers, and other aggregations (i.e., clusters, ≥ 3 particles) in these samples. S: Single, D: Dimer, C: Cluster.

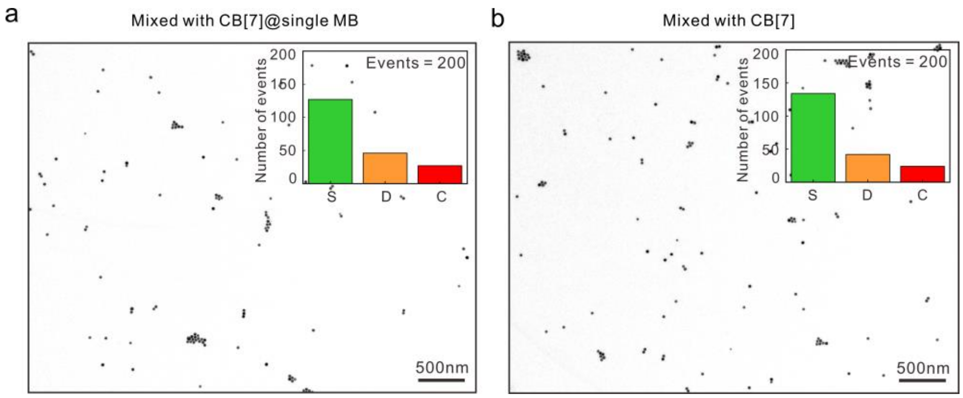

Supplementary Figure 6. Morphology characterization of the Au nanoparticles after treated by CB[7] and CB[7]@single MB emitters respectively. a TEM image of the Au nanoparticles mixed with CB[7]@single MB emitters. **b** TEM image of the Au nanoparticles mixed with CB[7] without MB molecules. The two samples were treated at the same CB[7] concentration of $\overline{n}_{CB[7]}$ ~1.6 CB[7] molecules per Au nanoparticle (on average). The insets are statistical histograms of the single Au nanoparticles, nanodimers, and other aggregations (i.e., nanoclusters, ≥ 3 nanoparticles). S: Single, D: Dimer, C: Cluster, MB: methylene-blue.

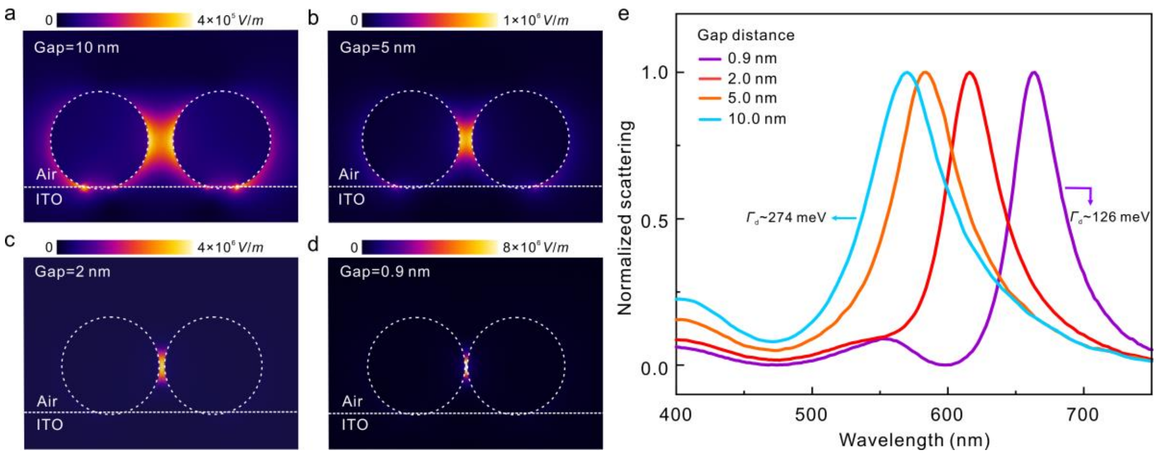

Supplementary Figure 7. EF distributions and scattering spectra of the AuNDs with different gap distances. a-d The EF distributions of AuNDs with different gap distances of (**a**) *d* = 10, (b) *d* = 5, (b) *d* = 2, and (d) *d* = 0.9 nm, respectively. **e** Normalized scattering spectra of the AuNDs localized on ITO-coated glass substrate with the gap distances of 10, 5, 2, and 0.9 nm, respectively, which were calculated using the FEM.

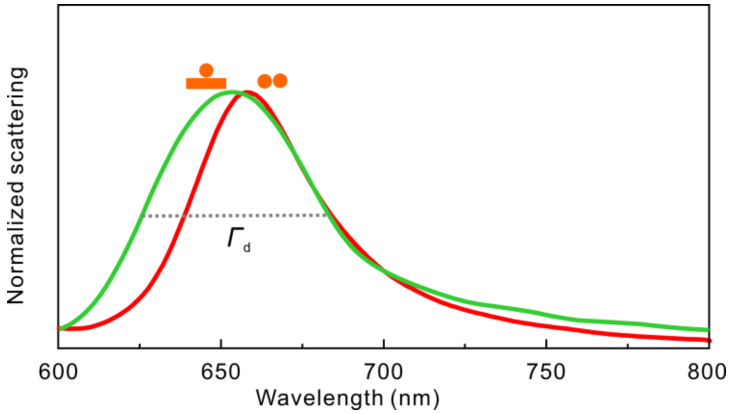

Supplementary Figure 8. Comparison of the simulated damping linewidths of the NPoM and AuND constructions. The green curve is the simulated scattering spectrum of the NPoM construct (see the inset, i.e., a 40-nm gold nanosphere localized on the gold nanofilm with a 0.9-nm gap), which is extracted from Supplementary Fig. 5 in the ref. 1); the red curve is the simulated scattering spectrum of the AuND composed by two 40-nm diameter gold nanospheres with a 0.9-nm gap.

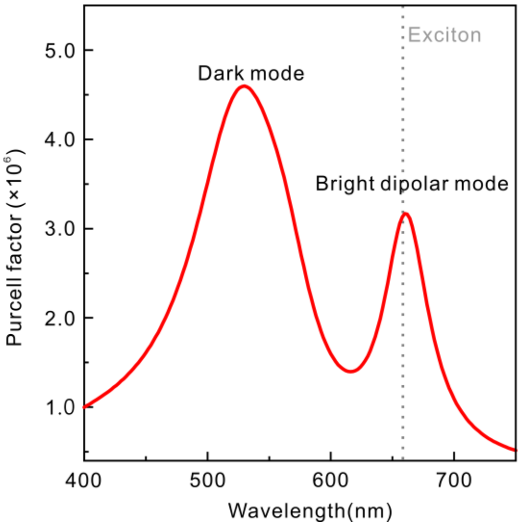

Supplementary Figure 9. Purcell factor of the AuND with a gap distance of *d* = 0.9 nm. Purcell factor simulation of the AuND constructed by a single CB[7] molecule (d = 0.9 nm), with a classical emitter placed in the center of the gap at the position of maximum field and spontaneous emission rate plotted as a function of wavelength.

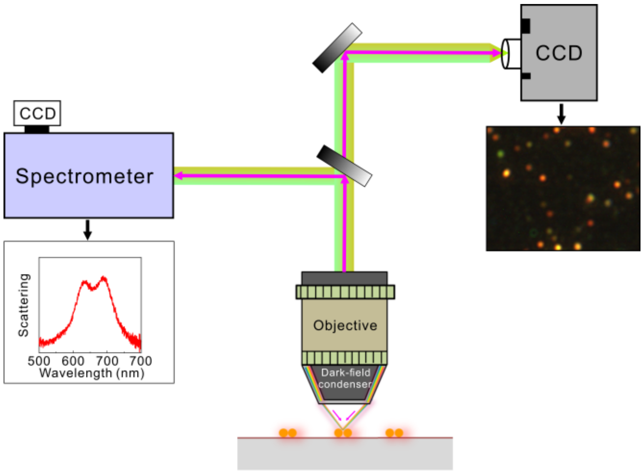

Supplementary Figure 10. Experimental system configuration for dark-field scattering measurements. Note that, for the collection of the scattering spectra, the light was launched from a dark-field objective (100×, numerical aperture 0.80), and the light scattered in the backward direction was collected by the same objective.

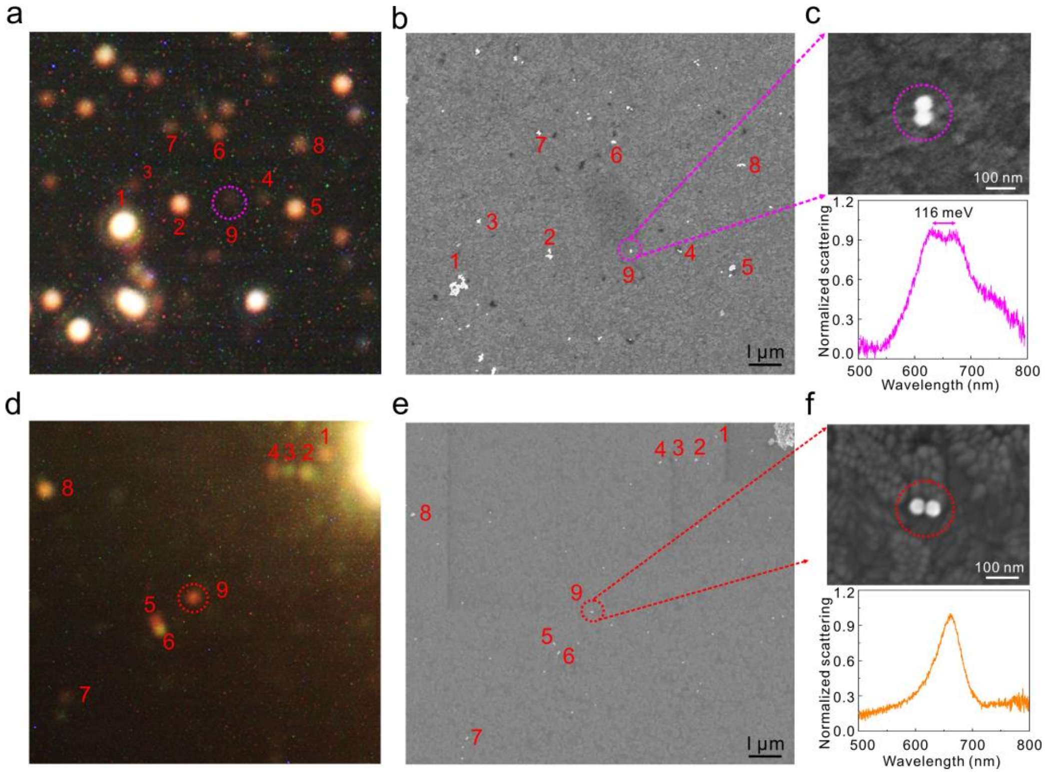

Supplementary Figure 11. Dark-field scattering images and the corresponding SEM images of the measured samples. a Dark-field scattering images of the measured AuND/CB[7]@single MB hybrids. **b** The corresponding SEM images of the measured AuND/CB[7]@single MB

hybrids in (**a**). **c** The corresponding SEM image in a larger magnification (upper panel) and the scattering measurement result (lower panel) for the AuND/CB[7]@single MB hybrid marked in (**b**). **d** Dark-field scattering images of the measured AuND/CB[7] without MB molecules. **e** The corresponding SEM images of the measured AuND/CB[7] without MB. **f** The corresponding SEM image in a larger magnification (upper panel) and the scattering measurement result (lower panel) for the AuND/CB[7] marked in (**d**).

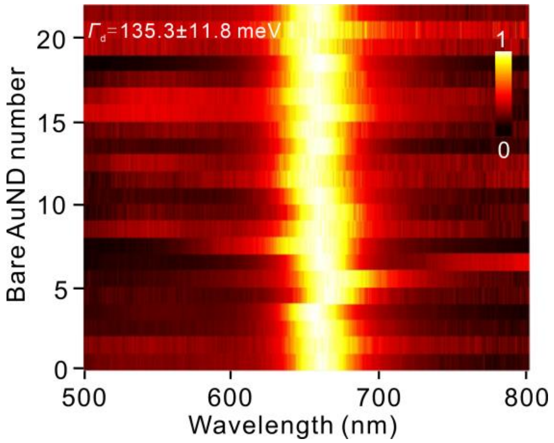

Supplementary Figure 12. Statistics of the damping linewidths for the bare AuNDs resonant to the exciton transition. Representative scattering spectra of the individual AuND/single CB[7] with the resonance wavelength at ~660-670 nm. A mean value of Γ_d = 135.3 ± 11.8 meV can be obtained.

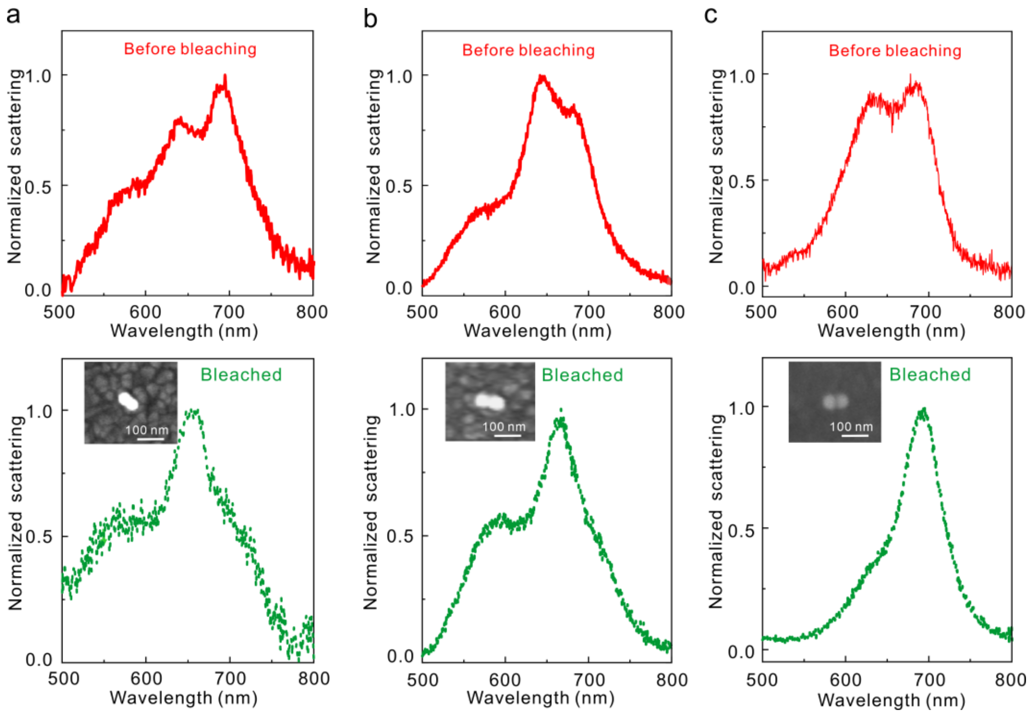

Supplementary Figure 13. Photobleaching of the strongly coupled AuNDs with MB-molecule excitons. **a-c** Scattering spectra of individual AuND/CB[7]@MB hybrids before (upper panels) and after (lower panels) photobleaching induced by laser illumination. Insets show SEM images of the measured hybrid AuNDs.

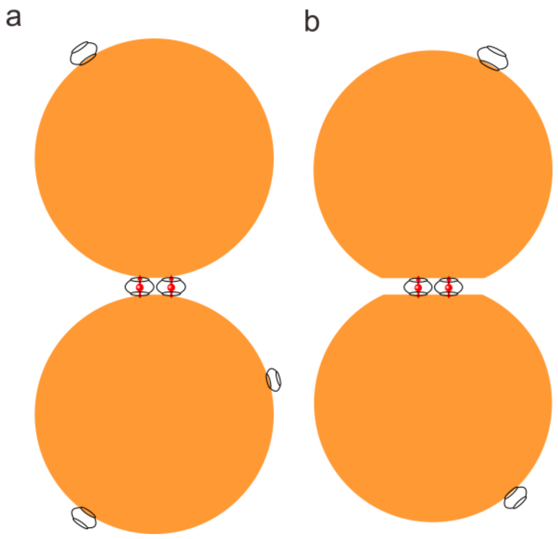

Supplementary Figure 14. Possibilities of two CB[7]@single MB emitters simultaneously accommodate in the gap of an AuND with and without facets at a low dye concentration of

$\overline{n}_{MB} = 1$ **CB[7]@single MB emitter and** $\overline{n}_0 = 1$ **empty CB[7] per Au nanoparticle on average**. **a** Schematic of CB[7]@single MB emitters and empty CB[7] molecules bound on an AuND constructed by two perfect Au spherical nanoparticles. The possibility for two CB[7]@single MB emitters embedded in the gap of the nanodimer is $p = 50\% \times (C_3^1 \times 1/3 \times 28.3/5024) \approx 0.28\%$. **b** Schematic of CB[7]@single MB emitters and empty CB[7] molecules bound on an AuND constructed by two Au nanoparticles with small facets. The possibility for two CB[7]@single MB emitters embedded in the gap of the nanodimer is $p = 50\% \times (C_3^1 \times 1/3 \times 78.5/5024) \approx 0.78\%$. It can be seen that the small facets of Au nanoparticles have a very limited influence on the single molecule accommodation in the AuND.

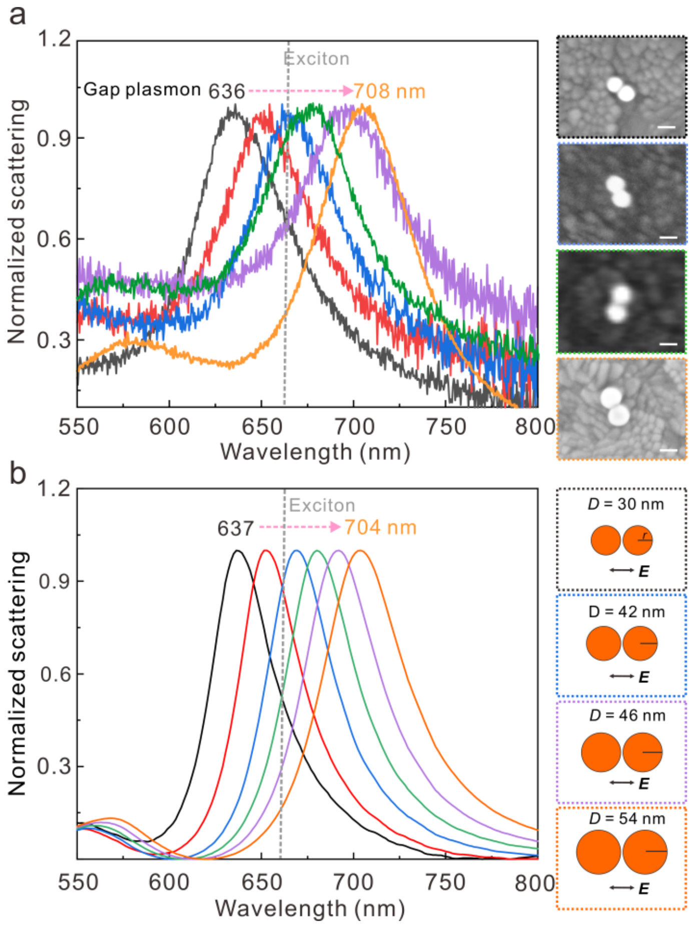

Supplementary Figure 15. Experimental and simulated scattering spectra of the bare AuNDs with different particle sizes. a Normalized scattering spectra of individual bare AuNDs with different particle sizes located on ITO-coated glass substrate show the plasmon resonance

wavelength varying from 636 to 708 nm. The right panel gives the SEM images of the measured samples related to the scattering spectra shown in the left panel with the same line color as the dotted box around the SEM images. The scale bar is 50 nm. **b** Normalized scattering spectra of the bare AuNDs (with d = 0.9 nm) located on ITO-coated glass substrate as a function of the particle size (diameter, D) varying from D = 30 to 54 nm, which were calculated using the FEM. The right panel gives the schemes of the calculated AuND structures related to the scattering spectra shown in the left panel with the same line color as the dotted box around these schemes.

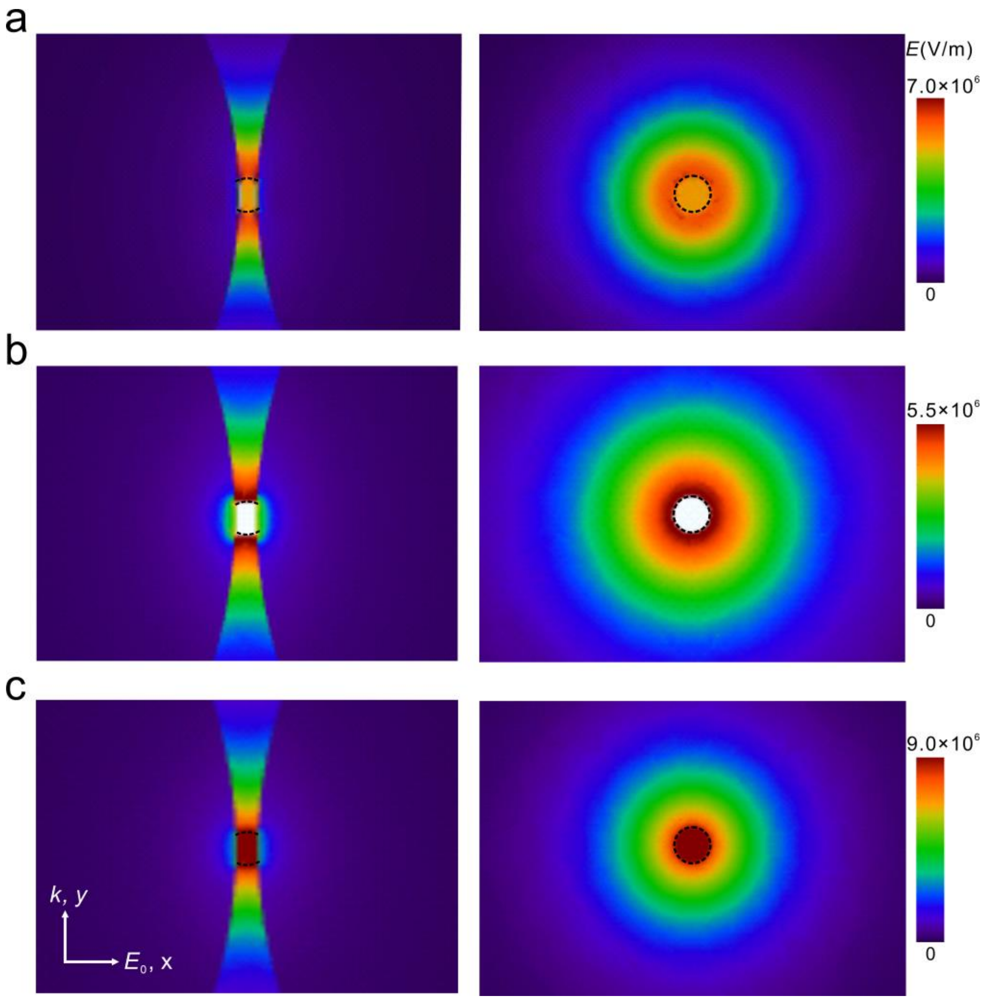

Supplementary Figure 16. Simulated near-field distributions in the gap of a strongly coupled AuND/CB[7]@single MB hybrid. Simulated near-field distribution map at (**a**) ω_+ (649 nm), (**b**) ω_c (665 nm), and (**c**) ω_- (692 nm) of the hybrid AuND/CB[7]@single MB. Left panel: In-plane parallel EF distributions in the middle of the gap. Right panel: in-plane vertical EF distributions in the center of the nanogap.

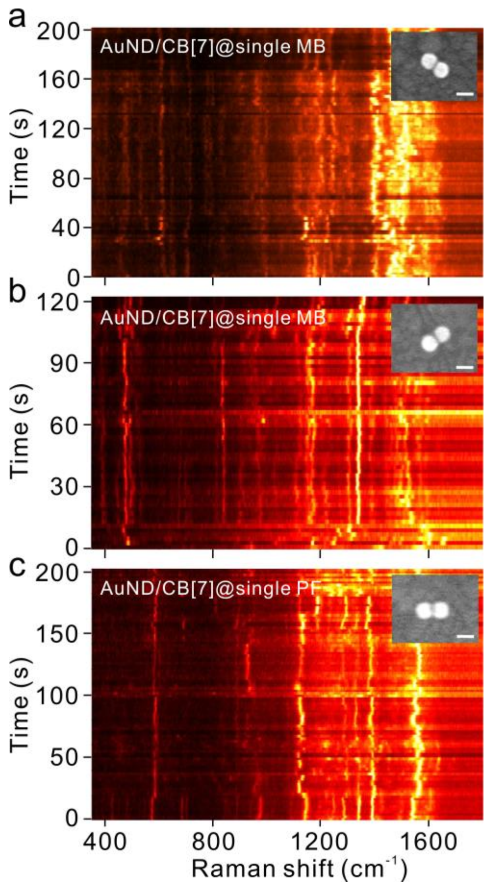

Supplementary Figure 17. Spectral diffusion of single-molecule SERS from different AuND. **a**, **b** Time-dependent evolution of SERS signals from different strongly coupled AuND/CB[7]@single MB hybrids. **c** Time-dependent evolution of SERS signals from an AuND/CB[7]@single PF hybrid. Each spectrum is collected for 2s at 633 nm. Insets are the SEM images of the measured hybrid AuNDs. The scale bar is 50 nm. AuND: Au nanodimer, MB: methylene-blue, PF: proflavine.

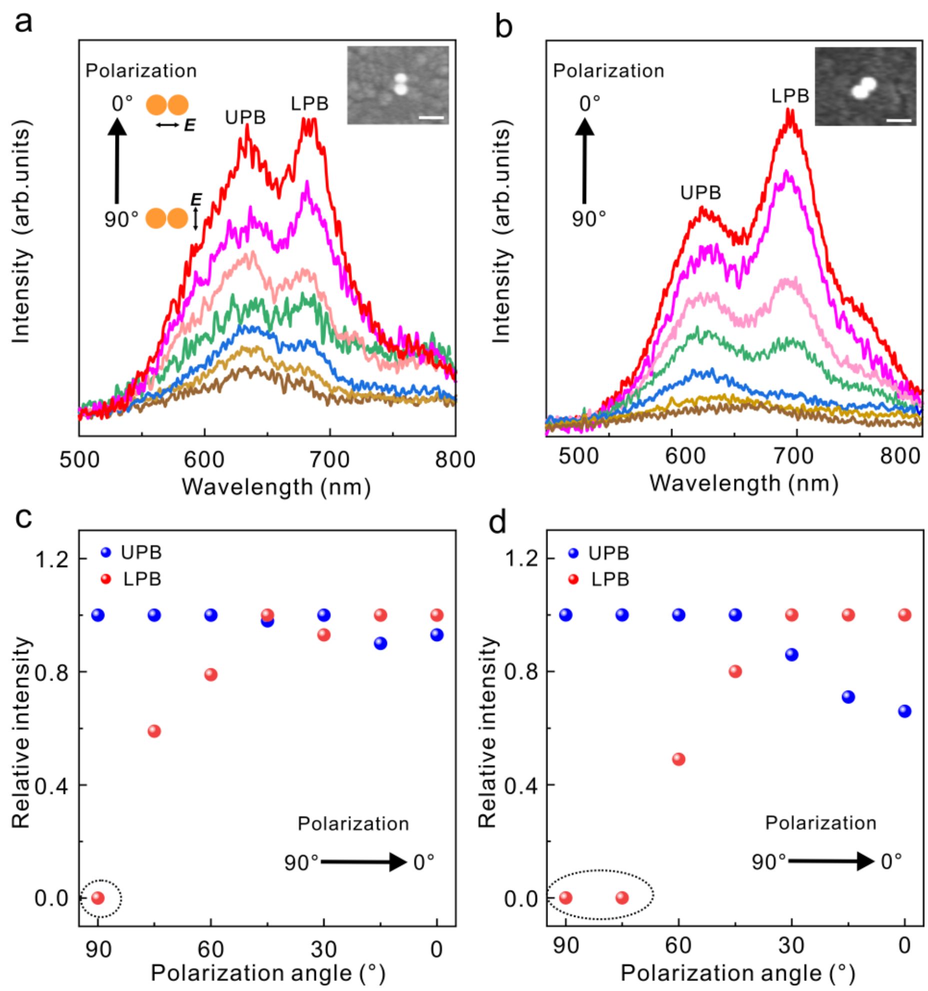

Supplementary Figure 18. **Polarization dependence of plexcitonic properties on a single hybrid AuND strongly coupled with MB-molecule excitons**. **a**, **b** Polarized scattering spectra for two cases of the strongly coupled AuNDs with MB-molecule excitons, which were detected at polarization angles of 0°, 15°, 30°, 45°, 60°, 75°, and 90°, respectively. The scale bar is 100 nm. **c**, **d** Relative scattering intensity of the UPB (blue balls) and LPB (red balls) modes as a function of the polarization angle for the two cases in (**a)** and (**b)**. Each polarized spectrum was normalized to its maximum scattering amplitude to compare the relative amplitudes of the UPB and LPB modes. The dashed circles in (**c**) and (**d**) indicate that the LPBs vanish at these polarization angles. UPB: upper plexciton branch, LPB: lower plexciton branch.

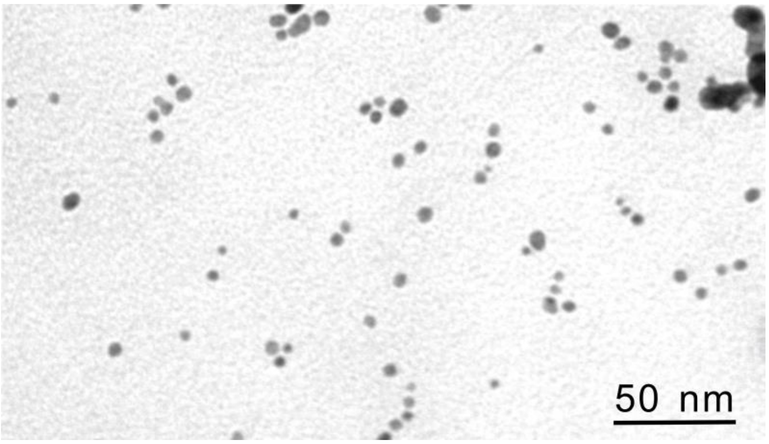

Supplementary Figure 19. TEM image of the Au nanoseeds.

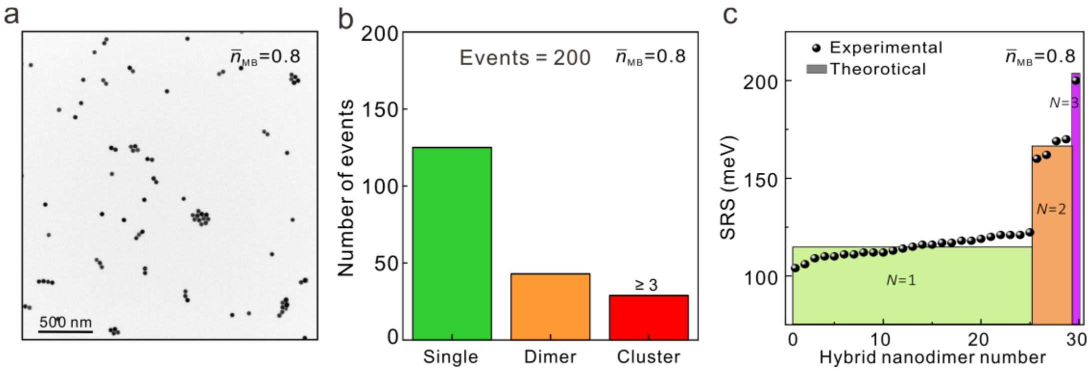

Supplementary Figure 20. Statistics of the AuNDs in the strong coupling. a Representative TEM images of the Au nanoparticles mixed with CB[7]@single MB emitters (at a concentration of $\bar{n}_{MB}$ ~0.8 CB[7]@single MB emitter per Au nanoparticle) for 1h. **b** Statistical histograms of the single Au nanoparticles, nanodimers, and other aggregations (i.e., clusters, ≥3 nanoparticles). **c** Quantum steps of the coupling strength observed from the strongly coupled hybrid AuNDs isolated from the Au nanoparticle ensemble treated with CB[7]@single MB emitters at a concentration of $\bar{n}_{MB}$ ~0.8 CB[7]@single MB emitter per Au nanoparticle. MB: methylene-blue, PF: proflavine.

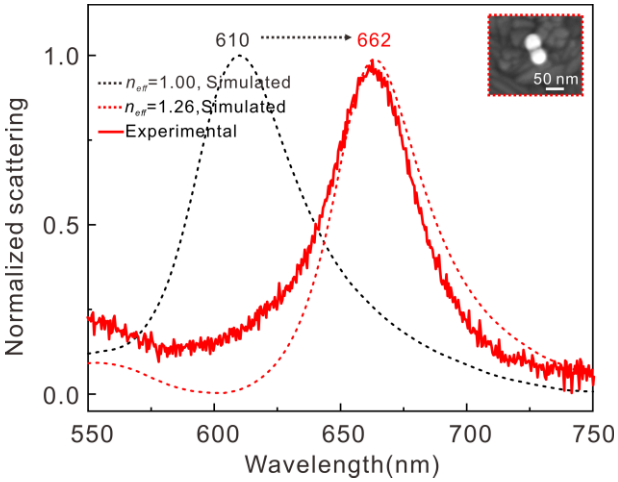

Supplementary Figure 21. **Experimental and simulated scattering spectra of the bare AuND.** The normalized experimental scattering (red curve) of an AuND on the ITO-coated glass substrate. The dashed red curve is the simulated scattering of the AuND located on the ITO-coated glass substrate using an effective refractive index n_{eff} = 1.26 above and around the ND. The dashed black curve is the simulated scattering of this AuND using the refractive index of air (n_{eff} = 1.00) above the substrate.

Supplementary Tables

Supplementary Table 1. Summary of emitter number (*N*) and emitter status (position and dipole direction) involved in strong couplings in the various plasmonic nanocavities.

**Strongly coupled systems**	**Coupling strength (meV)**	**Exciton materials**	**Minimum emitter number, *N***	**Emitter position certainty**	**Dipole direction certainty**
Single gold nanorod on monolayer WS_2[18]	~91	WS_2 monolayer	~5	×	×
A few dye molecules in the Au nanodimer[11]	~150	ATTO647N molecules	~5	√	×
Single Au dimer nanoantenna on monolayer WS_2[19]	~115.2	WS_2 monolayer	~4.67	×	×
Monolayer WS_2 in Au NPoM[20]	82	WS_2 monolayer	~4	×	×
Single MB molecules in Au NPoM[1]	~120	MB molecules	~2	×	√
J-aggregates covered a single Au@Ag NR[5]	~102	(PIC) J-aggregates	~1.38	×	×
Single QDs in silver bowtie plasmonic cavity[21]	120	Single CdSe/ZnS QD	1	√	×
A single QD under an Au nanotip[22]	160	Single CdSe/ZnS QD	1	√	×
A Single QD under a plasmonic nanoresonator[23]	110	Single CdSeTe/ZnS QD	1	√	×
A single QD in Au NPoM[16]	230	Single CdSe/CdS QD	1	√	×
A single QD integrated into a wedge nanogap cavity[24]	234	Single CdSe/ZnS QD	1	√	×
F-P cavity-engineered Au@Ag NR/ J-aggregates[25]	~62	(PIC) J-aggregates	~1	×	×
A single MB molecule in the AuND (This work)	116	A single-molecule exciton	1	√	√

Notice that, due to the multiple excitons generated in a single colloidal QD under the photoexcitation, the single QD strong coupling with plasmons here doesn't mean that there is a single exciton strongly coupling with plasmons. MB: methylene blue. It also should be mentioned that some debates exist on evaluating the exciton numbers involved in strong coupling by using the excitonic materials of J-aggregates or transition-metal dichalcogenide (TMD) monolayers[17].

Supplementary Table 2. Calculated V_m and g_0 for the AuNDs with different gap distances.

d	λ_{d} (nm)	Γ_{d} (meV)	V_m (nm^3)	V_{eff} (nm^3)	g_0 (meV)
0.9	661	130	26	42.6	56.9
2.0	610	151	90	147.5	31.8
5.0	576	192	472	773.4	14.3
8.0	565	210	780	1278	11.2
10.0	560	228	951	1558.2	10.2

In calculations, the diameter of the Au nanoparticle in the AuND is 40 nm, the nanodimer was located on the ITO-coated glass substrate, and the dipole moment $\boldsymbol{\mu}_{\mathrm{c}}$ of a single exciton was set at 0.09 e nm. The effective refractive index above the ITO substrate is set at 1.26 to ensure the calculated resonant wavelength of the AuND agrees with the experimental measurement results under the same structural parameters.

Supplementary References

1. Chikkaraddy, R. et al. Single-molecule strong coupling at room temperature in plasmonic nanocavities. *Nature* **535**, 127–130 (2016).

2. Nau, W. M. & Mohanty, J. Taming fluorescent dyes with cucurbituril. *Int. J. Photoenergy* **7**, 133−141 (2005).

3. Mohanty, J., Choudhury, S. D., Upadhyaya, H. P., Bhasikuttan, A. C. & Pal, H. Control of the supramolecular excimer formation of thioflavin T within a cucurbit[8] uril host: a fluorescence on/off mechanism, *Chem. Eur. J.* **15**, 5215–5219 (2009).

4. Zengin, G. et al. Realizing Strong light-matter interactions between single-nanoparticle plasmons and molecular excitons at ambient conditions, *Phys. Rev. Lett*. **114**, 157401 (2015).

5. Liu, R. et al. Strong light-matter interactions in single open plasmonic nanocavities at the quantum optics limit. *Phys. Rev. Lett.* **118**, 237401 (2017).

6. Rousseaux, B., Baranov, D. G., Käll, M., Shegai, T., & Johansson, G. Quantum description and emergence of nonlinearities in strongly coupled single-emitter nanoantenna systems. *Phys. Rev. B* **98**, 045435 (2018).
7. Rousseaux, B., Baranov, D. G., Antosiewicz, T. J., Shegai, T. & Johansson, G. Strong coupling as an interplay of quantum emitter hybridization with plasmonic dark and bright modes. *Phys. Rev. Research* **2**, 033056 (2020).
8. Liao, Z., Lu, Y., & Wang, X. -H. Optical scattering imaging with sub-nanometer precision based on position-ultra-sensitive giant Lamb shift. *arXiv:2211.03247v2*.
9. Manjavacas, A., García de Abajo, F. J. & Nordlander, P. Quantum plexcitonics: strongly interacting plasmons and excitons, *Nano Lett*. 11, 2318–2323 (2011).
10. Zubarev, D. N. Double-time Green functions in statistical physics. *Sov. Phys. Usp*. **3**, 320 (1960).
11. Heintz, J., Markešević, N., Gayet, E. Y., Bonod, N. & Bidault, S. Few-molecule strong coupling with dimers of plasmonic nanoparticles assembled on DNA. *ACS Nano* **15**, 14732−14743 (2021).
12. Schlather, A. E., Large, N., Urban, A. S., Nordlander, P. & Halas, N. J. Near-field mediated plexcitonic coupling and giant Rabi splitting in individual metallic dimmers. *Nano Lett*. **13**, 3281−3286 (2013).
13. Liu, R. et al. On-demand shape and size purification of nanoparticle based on surface area. *Nanoscale* **6**, 13145–13153 (2014).
14. Millstone, J. E. et al. Iodide ions control seed-mediated growth of anisotropic gold nanoparticles. *Nano Lett*. **8**, 2656–2659 (2008).
15. O'Brien, M. N. et al. Uniform circular disks with synthetically tailorable diameters: Two-dimensional nanoparticles for plasmonics. *Nano Lett*. **15**, 1012–1017 (2015).
16. Leng, H., Szychowski, B., Daniel, M. –C. & Pelton, M. Strong coupling and induced transparency at room temperature with single quantum dots and gap plasmons. *Nat. Commun*. **9**, 4012 (2018).
17. Tserkezis, C. et al. On the applicability of quantum-optical concepts in strong-coupling nanophotonics. *Rep. Prog. Phys*. **83**, 082401 (2020).

18. Wen, J. et al. Room-temperature strong light-matter interaction with active control in single plasmonic nanorod coupled with two-dimensional atomic crystals. *Nano Lett.* **17**, 4689-4697 (2017).
19. Liu, L. et al. Plasmon-induced thermal tuning of few-exciton strong coupling in 2D atomic crystals. *Optica* **8**, 1416−1423 (2021).
20. Qin, J. et al. Revealing strong plasmon-exciton coupling between nanogap resonators and two-dimensional semiconductors at ambient conditions. *Phys. Rev. Lett.* **124,** 063902 (2020).
21. Santhosh, K., Bitton, O., Chuntonov, L. & Haran, G. Vacuum Rabi splitting in a plasmonic cavity at the single quantum emitter limit. *Nat. Commun.* **7**, 11823 (2016).
22. Park, K. –D. et al. Tip-enhanced strong coupling spectroscopy, imaging, and control of a single quantum emitter. *Sci. Adv.* **5**, aav593 (2019).
23. Groß, H., Hamm, J. M., Tufarelli, T., Hess, O. & Hecht, B. Near-field strong coupling of single quantum dots. *Sci. Adv.* **4**, 4906 (2018).
24. Li, J. -Y. et al. Room-temperature strong coupling between a single quantum dot and a single plasmonic nanoparticle. *Nano Lett*. **22**, 4686−4693 (2022).
25. Li, W, et al. Highly efficient single-exciton strong coupling with plasmons by lowering critical interaction strength at an exceptional point. *Phys. Rev. Lett*. **130**, 143601 (2023).
