# Supplementary material for: Deterministic positioning and alignment of a single-molecule exciton in plasmonic nanodimer for strong coupling
Source: Nat Commun. 2024 May 16;15:4103. doi: 10.1038/s41467-024-46831-6 (PMC11099047; doi:10.1038/s41467-024-46831-6)
Supplement: Supplementary file 3 — Description of Additional Supplementary Files [file 41467_2024_46831_MOESM3_ESM.pdf]

Description of Additional Supplementary Files

Supplementary Movie 1: Movie of constructing an AuND/CB[7]@single MB hybrid.
